# Dentistry and Business

**Published:** 1921-09

**Authors:** 


### Dentistry and Business

In the March Journal I mentioned certain dental magazines and their work for business-like methods of educating the public.

In this connection two recent articles by Herbert G. Frenkel, D.D.S., are of interest—"Dentistry, What's the Matter with You?" in Oral Hygiene for June, and "Salesmanship in Dentistry," in The Dental Register for July.

The following paragraphs are condensed from the former article:

We have heard the wail, "The laity do not appreciate good dentistry and I am wasting my time and life."

I believe that the individual is part to blame and the colleges are to blame as well.

In dental school, he is taught how to do good work, but is left entirely upon his own resources as to salesmanship. He is not taught values, but workmanship. How many of the colleges teach their students to sell health rather than mechanics?

Salesmanship in dentistry is as important as any branch of the profession. All big corporations and industries have adopted the plan of calling their salesmen to conferences, where classes in salesmanship are given.

I believe that a class in salesmanship should be a part of the curriculum of every first-class dental school.

The dental surgeon should have business knowledge as well as professional knowledge.

It behooves us to try to correct the young men coming into the profession, so that they will not have to learn through bitter experience the things that we have suffered.

Is your office clean, bright and inviting, a place where sanitary operations can be performed, or dirty, dark, gloomy, bloody, with no attempts at sanitation?

Are you thoughtful, courteous, kind, with a great deal of patience, or do you appear slovenly, curt, cruel, impatient?

Can you convince your patients by salesmanship what is best for them and by skillful demonstrations show why your value is worth what you ask?

Do you read professional journals and do you follow the teachers of the profession?

Are you putting health before money insofar as to advise a patient? Do you instruct all your patients on the value of prophylaxis as compared with their health?

Are you treating your patients with the same regard that **you** would want to be treated?

If you are not doing these things, you can not say it is your profession that is wrong. **It is you!**—*Journal of Osteopathy.*

